# Genetic polymorphism rs3760396 of the chemokine (C-C motif) ligand 2 gene (CCL2) associated with the susceptibility of lung cancer in a pathological subtype-specific manner in Han-ancestry Chinese: a case control study

**DOI:** 10.1186/s12885-016-2328-8

**Published:** 2016-05-04

**Authors:** Xu Li, Fangcai Lin, Hong Zhou

**Affiliations:** Department of Pulmonary Medicine, Capital Medical University Electric Power Teaching Hospital, Taipingxili Jia 1, Beijing, 100073 China; Department of General Surgery, Capital Medical University Electric Power Teaching Hospital, Taipingxili Jia 1, Beijing, 100073 China

**Keywords:** CCL2, SNP, Genetic association, Lung cancer, Chinese population

## Abstract

**Background:**

Chemokines are well known inflammatory factors critical for tumor development in diverse tissues, including lung cancer. Chemokine (C-C motif) Ligand 2 (CCL2) was one of such chemokines important for both primary tumor development and metastasis of various cancers. Polymorphism at rs3760396 of CCL2 genes is associated with the prognosis of non-small cell lung cancer (NSCLC). The goal of our study was to examine the relationship of genetic polymorphisms rs3760396 with the susceptibility of lung cancer and its pathological subtypes in Han-ancestry Chinese population.

**Methods:**

rs3760396 G/C polymorphism of CCL2 was genotyped using PCR in 394 patients with lung cancer and 545 cancer-free controls from the same Northeast region of China.

**Results:**

After controlling for gender, age and smoking status, no significant association was observed between rs3760396 polymorphism and overall lung cancer. However, minor allele G of rs3760396 polymorphism was significantly associated with increased risk of adenosquamous lung carcinoma with either allelic genetic model (OR = 5.29, *P* < 0.001), or dominant genetic model (OR = 9.88, *P* < 0.001), or genotypic model (GC genotype vs. CC genotype, OR = 10.73, *P* < 0.001). Although rs3760396 polymorphism was not significantly associated with increased risk of adenocarcinoma subtype, it was nominally associated with the pooled outcome of either adenocarcinoma or adenosquamous carcinoma under allelic genetic model (OR = 1.54, *P* = 0.023) or dominant genetic model (OR = 1.57, *P* = 0.031).

**Conclusions:**

Our study suggested rs3760396 polymorphism of CCL2 is associated not only with prognosis of NSCLC, but also with risk of lung cancer in a subtype-specific manner. Our results further supported previous evidence of the important role of CCL2 in lung cancer development.

## Background

Globally, Lung cancer is the most common cancer diagnosed and is responsible for one fifth of death due to cancer [1]. In China, there are 0.7 million incidence cases, and the mortality caused by lung cancer is up to 0.6 million cases [2]. Most lung cancer patients are diagnosed at later stage when the timing for effective surgical dissection is missed. Its prognosis is relatively poor with 5-year survival rate at only about 15 % if diagnosed at later stage. While, the 5-year survival rate can be 30–40 % if diagnosed at early stage [3]. It has been found that both genetic and environmental factors are critical for the development of lung cancer. Although tobacco smoking can account for up to 80 % of lung cancer cases, substantial variations exist that cannot be explained solely by environmental and behavior factors [4]. Identification of genetic risk factors such as genetic polymorphism can have important indication for both early detection and therapy target discovery to improve the prognosis of lung cancer patients. Recent research has successfully identified potential genetic variations associated with the susceptibility of lung Cancer [5–8].

Inflammatory factors including cytokines and chemokines have long been suggested for their role in cancer development. Chemokines can contribute to tumor cell proliferation, angiogenesis and metastasis to promote the advancement of cancer [9]. Dysfunction of CXC and CC groups of chemokines has been found to involve in the progression of lung cancer [10, 11]. Chemokine (C-C motif) ligand 2 (CCL2), once named as monocyte chemotactic protein 1 (MCP-1), belongs to the CC chemokine family. CCL2 is primarily secreted by monocytes, macrophages and dendritic cells. It can recruit inflammatory cells to the sites of inflammation [12]. Polymorphism rs1024611 in CCL2 gene was reported to be correlated with the metastases of breast cancer and nasopharyngeal carcinoma [13, 14]. Very recently, another genetic polymorphism in the promoter region of CCL2 gene, rs3760396, has been reported to be associated with decreased risk of death for non-small cell lung cancer (NSCLC) in Chinese population [15]. Our current study sought out to study whether the same single nucleotide polymorphism (SNP: rs3760396) of CCL2 is also associated with the occurrence of lung Cancer and its pathological subtypes in a Chinese population.

## Methods

### Ethics, consent and permissions

The study was approved by the Ethical Committee of Capital Medical University Electric Power Teaching Hospital (Beijing, China). Consents to participate in the study from the participants (or legal guardian) were obtained.

### Consent to publish

We had obtained the consents to publish from the participant (or legal parent or guardian for children) to report individual patients’ data in any form (including images, videos, voice recordings etc.).

### Study subjects

Lung cancer patients were recruited from Capital Medical University Electric Power Teaching Hospital (Beijing, China) between Sep. 2011 to Sep. 2012. They were all newly diagnosed cases with histopathological confirmation. Lung cancer cases were classified histologically as squamous carcinomas, adenocarcinomas, adenosquamous carcinoma, small cell carcinomas, and large cell carcinomas. Patients would have been excluded if they had previous history of cancers, or history of chemotherapy/radiotherapy for other cancers. All control subjects are free of history of cancer or chemotherapy/radiotherapy.

### DNA extraction and genotyping assays

Peripheral blood samples were collected with EDTA tube and stored at −70 °C. DNA was purified from whole blood using the RelaxGene Blood DNA System (TianGen Biotech Co Ltd., Beijing, China) according to the manufacturer’s protocol. SNP rs3760396 located in gene chemokine (C-C motif) ligand 2 (CCL2) was genotyped using a Taqman SNP Genotyping Assay (Applied Biosystems, Foster City, CA, USA) with ABI 7900 HT Fast Real Time PCR System (Applied Biosystems). Its assay ID is C_27478341_10. Assays were performed with Taqman Universal Master Mix, Taqman probe, and 10 ng of DNA per reaction. PCR was set up according to manufacturer’s protocol: 3 min initial denaturation at 95 °C followed by 40 cycles of 95 °C denaturation for 15 s and 60 °C annealing/extension for 1 min. The genotyping process was performed blind to group status.

### Statistical analysis

Test of Hardy-Weinberg equilibrium of the genotype distribution was performed using exact tests implemented by Wigginton et al. [16] in PLINK 1.07 software. Characteristics of case and control groups were examined with student *t*-test or chi-squared test using STATA/SE12.0 (StataCorp LP, TX, USA). Logistic regression analysis was conducted to assess the association between the genotypes and overall lung cancer risk in PLINK 1.07 software and STATA/SE12.0 (StataCorp LP, TX, USA). A *p* value less than 0.05 was considered to be nominally significant. A total of 30 models involving up to six outcome traits and four types of genetic predictors have been tested. Under stringent Bonferroni correction on 30 tests, *P* value less than 0.0017 (=0.05/30) would be considered significant after correction on multiple testing. However, keep in mind that many outcome traits and predictors were related to each other. So this correction would be over-conservative. The association of genotypes with the pathohistological subtypes and various clinical stages of lung cancer were examined by multinomial logistic regression. Three subtypes were evaluated: squamous carcinomas, adenocarcinomas, adenosquamous carcinoma. Small cell carcinomas and large cell carcinomas were not included due to lack of variability of genotypes of rs3760396. Age and gender and smoking status were included as additive covariates in above analysis.

## Results

In current study, we genotyped CCL2 rs3760396 polymorphism in a total of 939 Han-ancestry Chinese subjects, including 395 patients with lung cancer and 545 healthy controls. One sample from case group without successful genotyping was excluded. Table 1 included the demographic and characteristics information of all the subjects. There were less males in cases versus controls (59.4 % vs. 67.3 %) (*p* = 0.012). Lung cancer patients were slightly older than controls (mean ± SD: 58.1 ± 9.4 vs 52.3 ± 10.5 years old, *p* <0.0001). There were significantly more smokers in cases (48.0 %) than in healthy controls (25.7 %) with a *p* value less than 0.001. Therefore, in the following SNP association analysis, age, gender and smoking status were included as covariates in an additive way.

**Table 1:** Characteristics of Lung Cancer Patients and Controls

Variable	Cases	Controls	*P* value^a^
Numbers	394	545	
Sex			0.012
Male	234	367	
Female	160	178	
Age (year)	58.1 ± 9.4	52.3 ± 10.5	< 0.0001
Han Ethnicities	394	545	
Smoking status			
Non-smokers	205	405	< 0.001
Smokers	189	140	
Histological type			
Squamous carcinomas	142	NA	
Adenocarcinomas	221	NA	
Adenosquamous carcinomas	20	NA	
Small cell carcinomas	10	NA	
Large cell carcinomas	1	NA	
TNM stages			
IA	10	NA	
IB	27	NA	
IIA	33	NA	
IIB	108	NA	
IIIA	73	NA	
IIIB	79	NA	
IV	63	NA	
not classified	1	NA	

^a^Two-sided *χ* ^*2*^ test

We summarized genotype distribution and allele frequency of rs3760396 SNP in CCL2 gene in controls and lung cancer cases (Table 2). rs3760396 SNP genotype distribution in both cases and controls was in agreement with the Hardy-Weinberg equilibrium (*p* = 0.40 and 0.53, respectively). G allele of rs3760396 SNP is the minor allele with higher prevalence in lung cancer patients than in controls. Its frequencies in controls and lung cancer patients were 7.5 and 9.8 %, respectively. Genotypes of rs3760396 SNP have a distribution of 0.7 %/13.6 %/85.7 % for GG/GC/CC genotypes in controls, and 1.3 %/17.0 %/81.7 % for GG/GC/CC genotypes in cases, respectively.

**Table 2:** Genotype and Allele Frequencies of the CCL2 rs3760396 polymorphism in lung cancer patients and controls

	Controls (%), *n* = 545	Cases (%), *n* = 394
Genotypes		
GG	4 (0.7 %)	5 (1.3 %)
GC	74 (13.6 %)	67 (17.0 %)
CC	467 (85.7 %)	322 (81.7 %)
Alleles		
G	7.5 %	9.8 %
C	92.5 %	90.2 %

*CCL2* chemokine (C-C motif) ligand 2 gene

We further tested the association of rs3760396 SNP with the susceptibility of lung cancer (Table 3). Without including any covariate, there is no significant genotypic or allelic association of rs3760396 SNP with lung cancer (*p* = 0.24 and *p* = 0.38, respectively). The association is non-significant either under dominant or recessive model (*p* = 0.10 and *p* = 0.41, respectively). Since age, gender and smoking status were significantly associated with lung cancer, we further included age, gender and smoking status as additive covariates in the association analysis. Four genetic models were evaluated. There was no significant association of this SNP with lung cancer although the point estimate of Odds Ratio (OR) showed the tendency of correlation between minor allele G and increased risk of lung cancer: OR = 1.25 and *p* = 0.2 in allelic model; OR = 1.24 and *p* = 0.26 in dominant model; OR = 2.07 and *p* = 0.31 in recessive model; and *p* = 0.4 in genotypic model.

**Table 3:** Statistical tests on the association of CCL2 rs3760396 polymorphism with lung cancer

Model	Comparison	Covariates	OR	*P*-value
Allelic	G vs C	none	1.35	0.38
Genotypic	GC vs GG vs CC	none	NA	0.24
Dominant	GG/GC vs CC	none	1.34	0.10
Recessive	GG vs GC/CC	none	1.74	0.41
Allelic	G vs C	Age, gender, smoking status	1.25	0.20
Genotypic	GC vs GG vs CC	Age, gender, smoking status	NA	0.40
Dominant	GG/GC vs CC	Age, gender, smoking status	1.24	0.26
Recessive	GG vs GC/CC	Age, gender, smoking status	2.07	0.31

*CCL2* chemokine (C-C motif) ligand 2 gene, *OR* odds ratio

Next, we examined the association of rs3760396 SNP with pathological subtypes of lung cancer. Small cell lung cancer and big cell lung cancer cases are all homozygous of the major allele C at rs3760396 SNP. Therefore, these two subtypes were not included in the analysis multinomial logistic regression was used. The results were summarized in Table 4. Because recessive genotypes were present in less than or equal to four cases in the subtypes of lung cancer, recessive genetic model was not evaluated. We found that rs3760396 SNP was significantly associated with adenosquamous carcinoma using either allelic (OR = 5.3 and *p* < 0.001), dominant (OR = 9.8 and *P* < 0.001), or genotypic model (OR = 10.7 and *P* < 0.001 for GC vs. CC genotypes) even under conservative Bonferroni correction (*p* value cutoff 0.0017). There is no significant association between rs3760396 and either adenocarcinoma or squamous cell carcinoma.

**Table 4:** Association of CCL2 rs3760396 polymorphism with pathological subtypes of lung cancer

Genetic model	Comparison	RRR^a^ vs. controls	*p*-value	95 % CI_Lower	95 % CI_Upper
Adenosquamous carcinoma					
allelic	G vs C	5.29	< 0.001*	2.40	11.66
Dominant	GC/GG vs CC	9.88	< 0.001*	3.61	27.00
genotypic	GC vs CC	10.73	< 0.001*	3.88	29.71
Adenocarcinoma					
allelic	G vs C	1.33	0.16	0.90	1.98
Dominant	GC/GG vs CC	1.29	0.25	0.84	1.99
genotypic	GC vs CC	1.22	0.39	0.78	1.90
	GG vs CC	2.89	0.16	0.66	12.62
Squamous cell carcinoma					
allelic	G vs C	0.82	0.48	0.47	1.43
Dominant	GC/GG vs CC	0.79	0.43	0.43	1.43
genotypic	GC vs CC	0.76	0.39	0.41	1.41
	GG vs CC	1.26	0.84	0.13	12.51

^a^ *RRR* relative risk ratio; *p < 0.05. *CCL2* chemokine (C-C motif) ligand 2 gene, *CI* confidence interval

We noticed that, the point estimates of OR for the association between the minor allele G of rs3760396 and adenocarcinoma is in the same direction as that in adenosquamous carcinoma, whereas the point estimate of OR was in opposite direction for squamous cell carcinoma. We speculated that rs3760396 polymorphism may be more associated with the adenocarcinoma cell components, which is shared between adenocarcinoma and adenosquamous carcinoma, than the squamous cell components in adenosquamous carcinoma. To test this hypothesis, we pooled the two pathological subtype of lung cancer together and examined the genetic association with Multinomial Logistic Regression. Again, age, geneder and smoking status were included as additive covariates. The results were presented in Table 5. Interestingly, nominally significant association remained between rs3760396 and the pooled pathological subtypes in which adenocarcinoma cell components were common (Relative Risk Ratio RRR = 1.54 and *P* = 0.02 in allelic model, and RRR = 1.57 and *p* = 0.03 in dominant model). Note that this association could not pass stringent Bonferroni corrected *p* value cut off (*p* < 0.0017).

**Table 5:** Association of CCL2 rs3760396 polymorphism with lung cancer cases classified as either adenocarcinoma or adenosquamous carcinoma

Genetic model	Comparison	RRR (vs Con)	*p*-value	95 % CI_Lower	95 % CI_Upper
adenocarcinoma/adenosquamous carcinoma					
allelic	G vs C	1.54	0.023	1.06	2.24
dominant	GC/GG vs CC	1.57	0.031	1.04	2.36
genotypic	GC vs CC	1.51	0.055	0.99	2.30
	GG vs CC	2.75	0.183	0.62	12.06
recessive	GG vs CC/GC	2.55	0.215	0.58	11.22

*CCL2* chemokine (C-C motif) ligand 2 gene, *RRR* relative risk ratio, *CI* confidence interval

We assessed whether the differential association of rs3760396 with pathological subtypes of lung cancers is mediated through differential distribution of clinical stages. Our analysis showed that adenosquamous cell carcinoma, adenocarcinoma and squamous cell carcinoma did exhibit differential distribution of clinical stages (chi-squared test *p* < 0.001). Therefore, we further included clinical stages as covariate. After controlling for clinical stages, SNP rs3760396 is still differentially associated with pathological subtypes using squamous cell carcinoma as reference (Table 6). In fact, we directly evaluated whether SNP rs3760396 is associated with the stages of lung cancer. Two analysis strategies were used: with control as reference outcome, there is no significant association of this SNP with any of the 4 stages of lung cancer (Results not shown); with case only analysis using stage 1 as reference outcome, there is no significant association of this SNP with stages of lung cancer either (Table 7). This provides another line of reasoning that our observed association of SNP rs3760396 with subtypes of lung cancer is not mediated through relationship with clinical stages.

**Table 6:** association of CCL2 rs3760396 polymorphism with pathological subtypes of lung cancer

Genetic model	Comparison	RRR	*p*-value	95 % CI_Lower	95 % CI_Upper
Squamous cell carcinoma		Reference Outcome			
Adenosquamous carcinoma					
allelic	G vs C	6.77	< 0.001*	2.58	17.74
Dominant	GC/GG vs CC	12.91	< 0.001*	4.10	40.61
genotypic	GC vs CC	14.42	< 0.001*	4.48	46.41
	GG vs CC				
Adenocarcinoma					
allelic	G vs C	1.73	0.09	0.93	3.24
Dominant	GC/GG vs CC	1.76	0.10	0.89	3.47
genotypic	GC vs CC	1.68	0.15	0.83	3.40

Note: Inclusion of clinical stages as a covariate in case only analysis on the association of CCL2 rs3760396 polymorphism with pathological subtypes of lung cancer. Squamous cell carcinoma was used as a reference subtype. *CCL2* chemokine (C-C motif) ligand 2 gene, *RRR* relative risk ratio, *CI* confidence interval

**Table 7:** Analysis on association of SNP rs3760396 with clinical stages of lung cancer within cases

Clinical stages	Genetic model	Comparison	RRR	*p*-value	95 % CI_Lower	95 % CI_Upper
1	Reference Stage					
2	allelic	G vs C	0.76	0.50	0.35	1.68
	genotypic	GC vs CC	0.55	0.19	0.23	1.35
	Dominant	GC/GG vs CC	0.63	0.30	0.26	1.51
3	allelic	G vs C	0.68	0.33	0.31	1.49
	genotypic	GC vs CC	0.52	0.15	0.22	1.27
	Dominant	GC/GG vs CC	0.57	0.21	0.24	1.37
4	allelic	G vs C	0.87	0.76	0.35	2.12
	genotypic	GC vs CC	0.81	0.67	0.30	2.14
	Dominant	GC/GG vs CC	0.82	0.70	0.31	2.18

*SNP* single nucleotide polymorphism, *RRR* relative risk ratio, *CI* confidence interval

## Discussion

Our current study found that the minor allele G of genetic polymorphism in CCL2 gene, rs3760396, was significantly associated with increased risk of adenosquamous lung carcinoma, after controlling for age, gender, and smoking status. The association remained nominally significant when adenosquamous lung carcinoma and adenocarcinoma were pooled together as a merged outcome phenotype, although the association *p* value could not pass over-conservative Bonferroni correction. This association was not observed for squamous cell lung carcinoma. There was no significant association of this SNP with overall lung cancer susceptibility, indicating subtype-specific effect of this SNP.

Furthermore, this subtype specific association is not confounded by the differential distribution of clinical stages among subtypes of lung cancer. In fact, there was no significant association between this SNP and clinical stages of lung cancer.

Our results pointed to a lung cancer subtype specific molecular mechanism link between CCL2 and adenosquamous carcinoma. To our best knowledge, our study represented the first such report. Our study further indicated the potential link of CCL2 rs3760396 with the adenocarcinoma cell components shared between adenosquamous carcinoma and adenocarcinoma. Our results extended recent finding in Chinese population that the same SNP is associated with the outcome of NSCLC [15]. Thus, the same genetic variation of CCL2 gene can affect both the risk of occurrence of adenosquamous cell lung carcinoma and the outcome of non-small cell lung cancer.

Interestingly, the association of CCL2 with adenocarcinoma or adenosquamous carcinoma in other tissue types has been reported. For example, recent study found increased expression of CCL2 mRNA in human gallbladder adenocarcinoma than those in human chronic cholecystitis [17]; Exogenous CCL2 expression by murine colon adenocarcinoma cells have been found to promote its lung metastases through promoting neovascularization [18, 19]. A pilot study [20] reported that CCL2 -2518A/G polymorphism is associated with genetic susceptibility to NSCLC in Han nationality of North China. rs3760396 has been rarely studied in lung cancer other than association between rs3760396 and decreased risk of death for non-small cell lung cancer [15]. A pilot case–control study in Chinese population [21] reported that the frequency of the heterozygote C/G at promoter of CCL2 was significantly less in ovarian cancer than in healthy controls.

The association between genetic polymorphism in CCL2 and lung cancer is biologically plausible. CCL2 plays an important role in the tumor microenvironment. CCL2 was reported as a transforming growth factor-β (TGF-β) target gene in endothelial cells (ECs). CCL2 mediates TGF-β–stimulated angiogenesis by enhancing migration of mural cells toward ECs and thus promoting the maturation of new blood vessels [22]. rs3760396 is located in the promoter region of CCL2 gene. It has been found that this SNP is associated with transcription factor binding sites and can modify transcriptional activity, and thus to be a functional SNP [23]. Previous studies revealed that CCL2 could regulate angiogenesis process in cancer development and metastasis through its critical role in macrophage recruitment [18]. Our current study together with other studies of rs3760396 [21] in Chinese population suggest that the G allele of rs3760396 in the promoter of CCL2 plays an important role in the function of CCL2. The exact mechanism underlying the link of rs3760396 of CCL2 with adenosquamous carcinoma would need further investigation.

Due to the limited sample size for small cell lung cancer and big cell lung cancer subtypes, we only evaluated the subtype specific effect of t rs3760396 in adenosquamous lung carcinoma, adenocarcinoma, and squamous cell carcinoma. It would warrant further investigation whether this SNP is also associated with increased risk of other pathological subtypes of lung cancer besides adenosquamous lung carcinoma. Future replication study is needed to confirm our finding and generalize our findings in other populations. Future functional study would be necessary to identify molecular mechanisms underlying the association.

## Conclusion

The minor allele of SNP rs3760396 of CCL2 gene is associated with increased risk of adenosquamous lung carcinoma, but not overall lung cancer in Chinese Han ethnicity population. The underlying functional mechanism would worth further investigation.

## Abbreviations

CCL2: chemokine (C-C motif) ligand 2
MCP-1: monocyte chemotactic protein 1
NSCLC: non-small cell lung cancer
OR: odds ratio
